# Longitudinal Relationships Between Organizational Justice, Productivity Loss, and Sickness Absence Among Older Employees

**DOI:** 10.1007/s12529-016-9546-y

**Published:** 2016-02-25

**Authors:** Jan F. Ybema, Laudry van der Meer, Fenna R. M. Leijten

**Affiliations:** 1 Social, Health and Organizational Psychology, Utrecht University, PO Box 80140, 3508 TC Utrecht, Netherlands; 2 TNO Work & Employment, Schipholweg 77-89, 2316 ZL Leiden, Netherlands; 3 Institute of Health Policy and Management, Erasmus University Rotterdam, PO Box 1738, 3000 DR Rotterdam, Netherlands

**Keywords:** Longitudinal study, Organizational justice, Productivity loss, Sickness absence, Older employees

## Abstract

**Purpose:**

The aim of this study was to assess whether organizational justice lowers productivity loss and sickness absence, and whether there are reverse effects of productivity loss and sickness absence on organizational justice.

**Method:**

A longitudinal study with 2 years of follow-up was conducted among employed persons aged 45–64 years from the Study on Transitions in Employment, Ability and Motivation (STREAM). Participants (*N* = 7011) yearly filled out an online questionnaire. Structural equation modeling in LISREL was conducted to assess the longitudinal relationships between distributive justice of salary, distributive justice of appreciation, procedural justice, productivity loss, and sickness absence.

**Results:**

Both distributive justice of appreciation and procedural justice contributed to lower productivity loss and lower sickness absence at 1-year follow-up. Productivity loss increased perceptions of distributive justice of appreciation at 1-year follow-up, whereas sickness absence lowered both perceptions of distributive justice of appreciation and procedural justice at follow-up.

**Conclusion:**

Improving organizational justice lowers the risk of productivity loss and sickness absence and may be a useful tool to improve the productivity of organizations.

## Introduction

Due to the ageing workforce and decreasing birth rates, organizations increasingly rely on older employees. Therefore, it is important for these organizations that their older employees remain healthy and work productively. In this study, we examine whether organizations can reduce productivity loss and sickness absence among older employees by improving the way they are treated. If organizations treat their employees in a just way this signals that all employees are valued [1], which may improve the health of employees and the functioning of the organization. Systematic reviews of prospective studies showed that organizational justice improves employees’ mental health [2] and protect against coronary heart disease [3].

Organizational justice can be divided into a distributive, procedural, and interactional component [4]. In the present study, we examine the distributive and the procedural components of organizational justice. Distributive justice has been defined as having an equal proportion between outcomes (e.g., salary, appreciation) and investments (e.g., work efforts, capacities) in comparison to colleagues [5]. Procedural justice refers to consistent and accurate procedures in decision making in which employees are treated ethically by the organization, and interests of employees are taken into account [6]. Both distributive and procedural justice also convey information about fair treatment by the supervisor, i.e., interactional justice [7, 8]; therefore, we decided not to separately include interactional justice in the current study.

In this longitudinal study with three yearly measurements among older employees, we examine how organizational justice is longitudinally related to productivity loss and sickness absence. We consider productivity loss while at work and sickness absence as behaviors on the continuum of withdrawal from work, which may or may not be due to health problems [9]. It should be noted that productivity loss and sickness absence cannot occur simultaneously. Nevertheless, as they share determinants, including health problems, productivity loss and sickness absence are generally positively related over time [9].

The first research question is how does organizational justice affect productivity loss and sickness absence? According to the “equity rule,” when people are rewarded in direct proportion to their individual contribution, they perform better [10–12] and they are less absent due to illness [13]. Procedural justice has been shown to improve job satisfaction, job commitment, and better health and well-being at work [2, 4, 13], which in turn may improve productivity and lower sickness absence [14–18]. It has to be noted that a meta-analysis found only modest relationships between organizational justice and performance or withdrawal behaviors [4], and not all studies find these relationships. For a more comprehensive review of relevant past research, we refer to Conlon and colleagues [19]. We did not find previous research on the relationship between organizational justice and *productivity loss*, which we consider a withdrawal behavior rather than a performance measure. The unique contribution of the present study is that we examine both withdrawal behaviors, i.e., productivity loss and sickness absence, in a single longitudinal study. The main hypotheses of the current study are as follows:

- *Hypothesis 1a*: Procedural and distributive justice lower productivity loss
- *Hypothesis 1b*: Procedural and distributive justice lower sickness absence

The second research question concerns possible reversed effects of productivity loss and sickness absence on organizational justice [13, 20, 21]. Unfair distributions of salary or lack of appreciation may lead workers to emotionally or physically withdraw from the workplace or invest less time and effort in their work, in an attempt to restore the equity between give and take [5]. If this strategy is effective, it could be expected that lowering productivity or becoming absent may increase perceived distributive justice (*restore equity*), albeit at a lower level. On the other hand, frequent or prolonged productivity loss or sickness absence may have negative consequences for relationships at work (*erosion*) and may lead to lower job satisfaction [22] and to lower distributive justice [13]. Therefore, it may also be expected that productivity loss and sickness absence lower distributive justice. At present, it is unclear which of these opposite processes, restore equity or erosion, will dominate. We therefore developed two opposite hypotheses regarding the reversed effects for both productivity loss and sickness absence. Evidently, these hypotheses (a and b) cannot be supported simultaneously:

- *Hypothesis 2a*: Productivity loss increases distributive justice (*restore equity*)
- *Hypothesis 2b*: Productivity loss decreases distributive justice (*erosion*)
- *Hypothesis 3a*: Sickness absence increases distributive justice (*restore equity*)
- *Hypothesis 3b*: Sickness absence decreases distributive justice (*erosion*)

For procedural justice, no reversed effects are hypothesized, because we have no evidence from previous studies that productivity loss or sickness absence would increase or decrease procedural justice. We therefore examine potential reversed effects on procedural justice in an exploratory fashion.

With the ageing workforce, older employees need to work longer than before as a result of the increasing retirement age and reduced possibilities for early retirement. Therefore, it is crucial to understand how older workers can remain in the workforce in a healthy and productive manner. The current study specifically examined the longitudinal relationships between organizational justice, productivity loss, and sickness absence among older employees in an attempt to support them and their organizations to remain productive for longer.

## Method

### Study Design and Sample

The Study on Transitions in Employment, Ability and Motivation (STREAM) is a Dutch prospective cohort study of employed persons, self-employed persons, and nonworking persons aged 45 to 64 years. STREAM measurements were carried out in October and November of 2010, 2011, 2012, and 2013 with online questionnaires among an existing Intomart Gfk internet panel. The online questionnaire contained questions on many topics, such as demographics, lifestyle, occupation (branch of industry, occupation, working hours), working conditions, sickness absence, psychological well-being and work satisfaction, and organizational culture.

At the time of the current study, data from the first three waves were available. At the baseline measurement in 2010, 12,055 employees completed their questionnaires, a response rate of 70 %. Of these employees at baseline, 9933 (82.4 %) participated in the second wave in 2011, 9632 (79,9 %) participated in the third wave in 2012, and 8752 (72,6 %) participated in all three measurements. In the present study, only persons who participated in all three measurements and who were employee at all three measurements were included (*n* = 7379). Furthermore, only persons with complete information on all relevant measures of the present study were included, resulting in a study sample of 7011 employees. More information on the design of STREAM can be found in Ybema et al. [23].

### Measures

#### Organizational Justice

The measures of distributive and procedural justice by De Boer et al. [14] and Ybema and Van den Bos [13] were used in this study. A confirmative factor analysis (CFA) on all nine organizational justice items at the baseline measurement resulted in a three-factor solution (i.e., distributive justice of salary, distributive justice of appreciation, and procedural justice). Based on this CFA, scales for distributive justice of salary (Cronbach’s α = 0.86), distributive justice of appreciation (Cronbach’s α = 0.88), and procedural justice (Cronbach’s α = 0.86) were constructed.

*Distributive justice of salary* was measured with three items, for example “What do think of your salary when you compare your work efforts with those of your colleagues?” *Distributive justice of appreciation* was measured with three similar questions concerning the appreciation for one’s work, for example “What do you think of the appreciation you get when you compare the number of tasks you have with those of your colleagues?” Answer options were as follows: 1 = *far too little salary*/*appreciation*, 2 = *somewhat too little*, 3 = *exactly right*, 4 = *somewhat too much*, and 5 = *far too much salary*/*appreciation*. As in previous research with similar measures [7, 13, 14], these answers were recoded to get a valid scale for both forms of distributive justice (1 = *far too little*/*far too much*, 2 = *somewhat too little*/*somewhat too much*, and 3 = *exactly right*) and were averaged to obtain scale scores. This means that these scales for distributive justice treat both over-benefit and under-benefit as forms of distributive injustice, although, in line with previous studies [7, 13], over-benefit was found less often than under-benefit.

*Procedural justice* was measured with three items that were primarily concerned with structural aspects of procedural justice [14]. The items were “The opinion of employees is taken into account,” “All employees are treated in a similar way,” and “Complaints of employees are taken seriously,” with answers ranging from 1 = *fully disagree* to 5 = *fully agree*. These items were averaged to obtain a scale score for procedural justice.

#### Productivity Loss

Productivity loss was measured with the single question: “How much work have you done in the last 4 weeks compared to normal?”. It was explained that only the days someone had worked in the last 4 weeks should be taken into account. The answers were measured on a scale ranging from 1 = *much less than normal*, through 6 = *as much as normal* , to 11 = *much more than normal*. As we were interested in productivity loss, the scale was recoded to a scale of 0 to 5. A score of 0 was allocated to individuals who had done a normal amount of work or had done more work than normal, and a score of 5 for those who had done much less work than normal. This measure was adapted from the QQ scale for productivity loss [24], which exclusively measures doing *less* work than normal. With our recode, we more or less return to the original QQ-scale.

#### Sickness Absence

Sickness absence was measured with a single question on how many days participants had been absent from work due to illness in the past 12 months, counting only days on which they usually worked. Based on the number of days a week participants indicated that they usually worked (also assessed in the questionnaire), this was recoded into an individual sickness absence percentage. This measure ranged from 0 % (no absence) to 100 % (absent all year).

### Analyses

The data were analyzed using SPSS for Windows and with the packages PRELIS 2 [25] and LISREL 8 [26]. In the SPSS analyses, changes over time in the variables of study were examined using analysis of variance for repeated measures. PRELIS was used for data screening and for the construction of covariance matrices that were used in LISREL. In the LISREL analyses, six nested models of the longitudinal relationships between organizational justice and productivity loss and sickness absence were tested, using a robust maximum likelihood (RML) method [27]. In RML, the standard errors of the parameter estimates are corrected for nonnormal distributions by using the asymptotic covariance matrix. Especially, the productivity loss and sickness absence measures were highly skewed and had a high kurtosis.

**Fig. 1:**
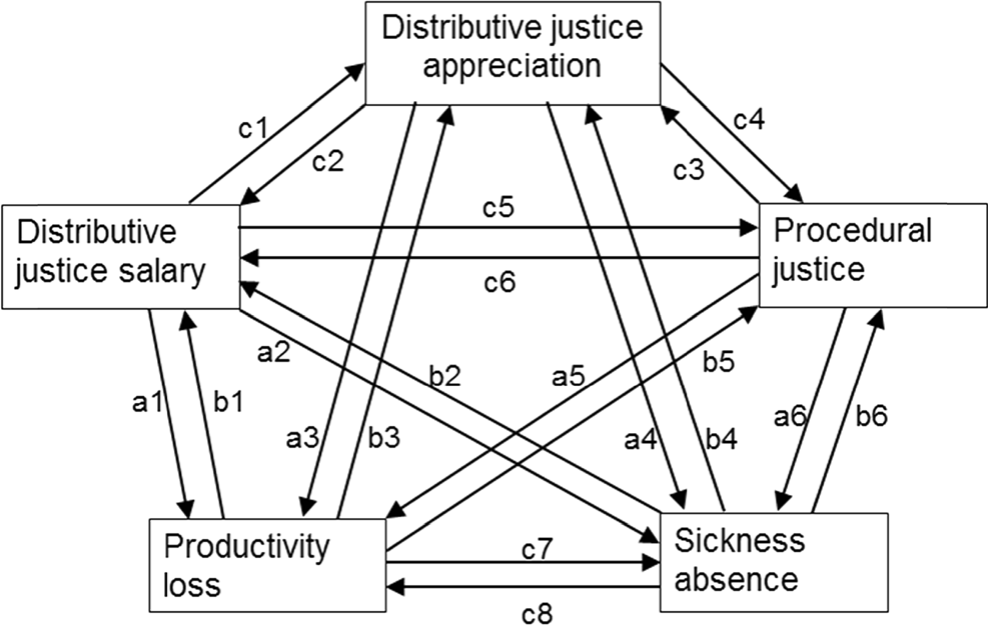
The pattern of longitudinal relationships between organizational justice, productivity loss, and sickness absence

The constructs were all analyzed as observed variables, leading to a path model rather than a structural equation model with latent variables. Figure 1 presents the longitudinal paths tested between the five endogenous variables: distributive justice of salary, distributive justice of appreciation, procedural justice, productivity loss, and sickness absence. Exogenous variables, i.e., gender, age, and education, were included in all models in order to explore how they related to organizational justice, productivity loss, and sickness absence and in order to control for potential confounding effects.

In the null model (M0), all exogenous and endogenous variables were considered as independent variables with no interrelationships. In M1, only the longitudinal paths of each endogenous variable on the same variable at the next measurements are estimated, which provides for information on the stability of the measures through time. In M2, in addition to the relationships in M1, the exploratory longitudinal relationships among the three organizational justice measures (paths c1 to c6) and among productivity loss and sickness absence (paths c7 and c8) were estimated. However, the longitudinal relationships between distributive and procedural justice on the one hand and productivity loss and sickness absence on the other hand (paths a1 to a6 and b1 to b6) were constrained to be zero in this model. In the normal causation model M3, six additional longitudinal relationships were estimated, those from the three organizational justice measures to productivity loss and sickness absence a year later (paths a1 to a6), whereas the reversed effects (b1 to b6) were constrained to be zero. Model M3 tests the effects of organizational justice on productivity loss and sickness absence (Research Question 1). In the reversed causation model M4, instead the six longitudinal relationships from productivity loss and sickness absence to the three organizational justice measures a year later were estimated (paths b1 to b6), whereas the normal causation paths (a1 to a6) were constrained to be zero. This model M4 tests the reversed effects of productivity loss and sickness absence on organizational justice (Research Question 2). In the reciprocal model M5, all longitudinal paths in Fig. 1 were estimated, thus including both the longitudinal effects of justice on productivity loss and sickness absence (a1 to a6; normal causation) and the longitudinal effects of productivity loss and absence on justice (b1 to b6; reversed causation).

In the models M1 to M5, the cross-sectional covariances (psi) within each measurement between distributive justice, procedural justice, productivity loss, and sickness absence were estimated and were constrained to be equal in all measurements. It has to be noted that these cross-sectional covariances are residual covariances which are corrected for the influence of the longitudinal relationships (stability over time and influence of other predictors). In addition, comparable longitudinal effects (beta) from T2 to T3 were constrained to be equal to those of T1 to T2. Finally, the effects of the demographic variables on all variables in the model (gamma) were constrained to be equal in all measurements. This was done because the cross-sectional and longitudinal paths that were constrained to be equal should be regarded as replications. There were no methodological or theoretical reasons why these parameters should be different. Moreover, these equality constraints resulted in a parsimonious model.

For evaluating the fit of the models, the Satorra Bentler chi-square for nonnormal distributions (*χ*^2^), the comparative fit index (CFI), the root mean square error of approximation (RMSEA) and the standardized root mean square residual (SRMR) were used. Values of 0.95 and above for CFI and values of 0.08 and below for RSMEA and SRMR indicate a good fit of the model [28].

## Results

### Descriptive Statistics and Change Over Time

In Table 1, the descriptive statistics for all variables in this study are presented. Of the 7011 respondents, 44 % were female and 56 % were male, with a mean age of 54 years at the first wave. The three measures of organizational justice were moderately high at all three waves. Most respondents had not experienced productivity loss in the last 4 weeks (93 %). The average sickness absence percentage was 4.6 % (SD = 13.4), which is slightly lower than sickness absence in the Dutch population of employees aged 45 to 64 years [29], and corresponds to approximately two workings weeks lost per year. In each measurement, more than half of the respondents (54 %) had not been absent due to sickness in the last 12 months.

**Table 1:** Descriptive statistics

	T1		T2		T3	
	%/M	SD	M	SD	M	SD
Gender (% female)	44 %					
Age (years)	53.59	5.14				
Education						
1 low	26 %					
2 moderate	39 %					
3 high	35 %					
Distributive justice salary (1–3)	2.42	0.59	2.40	0.61	2.42	0.60
Distributive justice appreciation (1–3)	2.55	0.56	2.54	0.58	2.54	0.58
Procedural justice (1–5)	3.29	0.82	3.25	0.83	3.23	0.83
Productivity loss (0–5)	0.20	0.85	0.20	0.85	0.24	0.94
Sickness absence (%)	4.43	12.65	4.61	13.67	4.70	13.79

Listwise deletion of missing values, *N* = 7011

Analyses of variance for repeated measures showed that distributive justice of salary was somewhat lower at the second measurement than at the first and third measurements, *F*(2, 7009) = 9.0, *p* < 0.001, distributive justice of appreciation did not change, *F*(2, 7009) = 2.5, *ns*, procedural justice lowered over time, *F*(2, 7009) = 23.0, *p* < 0.001, productivity loss was higher at the last measurement than at the first two measurements, *F*(2, 7009) = 6.4, *p* < 0.01, and sickness absence did not change significantly during the three waves, *F*(2, 7009) = 0.9, *ns*.

### Model Fit

In LISREL, six nested models were tested (see “Method” for details) to examine the cross-sectional and longitudinal relationships between organizational justice, productivity loss, and sickness absence. The fit of these six models are presented and compared in Table 2.

**Table 2:** LISREL model test and fit indices

		Sattora Bentler *χ* ^2^	*df*	*p* value	CFI	RMSEA	SRMR
M0	Null model	17,734	153	0.000	0.00	0.130	0.200
M1	Stability	1430	115	0.000	0.92	0.040	0.083
M2	Exploratory relations	669	107	0.000	0.96	0.027	0.055
M3	Normal causality	634	101	0.000	0.97	0.027	0.053
M4	Reversed causality	654	101	0.000	0.97	0.028	0.055
M5	Reciprocal model	620	95	0.000	0.97	0.028	0.052
		Δ*χ* ^2^		*p* <			
M3 vs M2	Normal causality	35	6	0.001			
M4 vs M2	Reversed causality	15	6	0.05			
M5 vs M2	Reciprocal model	49	12	0.001			
M5 vs M3	Reciprocal vs normal	14	6	0.05			
M5 vs M4	Reciprocal vs reversed	34	6	0.001			

**Fig. 2:**
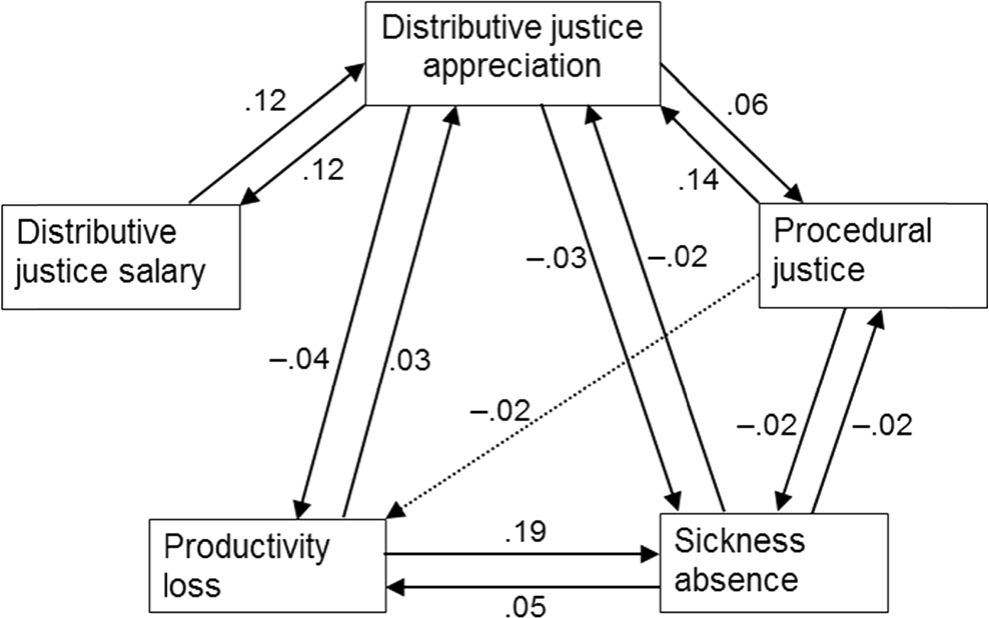
The longitudinal relationships between organizational justice, productivity loss, and sickness absence in the reciprocal model (M5), with standardized coefficients; *solid lines* are significant at *p* < 0.05, two-sided; *dashed line* is significant at *p* < 0.05, one-sided

The Satorra Bentler chi-square was significant for all tested models, but the other fit indices show that the latter four models (M2, M3, M4, and M5) had an acceptable fit. Nevertheless, the comparison of these four models shows that the normal causation model M3 had a much better fit than the exploratory relations model M2, and that the reversed causation model M4 also had a somewhat better fit than M2. Moreover, the reciprocal causation model M5 had a better fit than all three less inclusive models. Therefore, the longitudinal paths from the reciprocal causation model are discussed below. Figure 2 presents the longitudinal relationships in the final model M5. The (averaged) standardized parameters are given in Fig. 2 because they are easier to interpret, although the equality constraints concerned the unstandardized parameters, as is recommended in such models [26].

### Exploratory Longitudinal Paths

Figure 2 shows that distributive justice of salary was reciprocally related to distributive justice of appreciation (β = 0.12, *p* < 0.001, both ways). Both forms of distributive justice strengthened each other over time. A similar reciprocal longitudinal pattern was found for distributive justice of appreciation and procedural justice. In this longitudinal relationship, procedural justice enhanced distributive justice of appreciation a year later (β = 0.14, *p* < 0.001) to a stronger extent than the reversed effect of distributive justice of appreciation on procedural justice (β = 0.06, *p* < 0.001). There were no direct longitudinal relationships between procedural justice and distributive justice of salary.

The longitudinal relationship between productivity loss and sickness absence also showed a reciprocal pattern, in which sickness absence increased productivity loss in the following year (β = 0.05, *p* < 0.01). However, the reversed effect was stronger: productivity loss substantially increased sickness absence in the following year (β = 0.19, *p* < 0.001).

### Organizational Justice and Productivity Loss

Hypothesis 1a stated that organizational justice would lower productivity loss. Figure 2 shows that distributive justice of salary was not related to lower productivity loss (β = 0.02, *ns*). However, as predicted, both distributive justice of appreciation (β = −0.04, *p* < 0.01) and procedural justice (β = −0.02, *p* < 0.05, one-sided) contributed to lower productivity loss a year later.

No reversed longitudinal effects of productivity loss on distributive justice of salary (β = 0.02, *ns*) nor on procedural justice were found (β = 0.00, *ns*). However, productivity loss increased distributive justice of appreciation (β = 0.03, *p* < 0.05) a year later, which is in line with hypothesis 2a (*restore equity*).

**Fig. 3:**
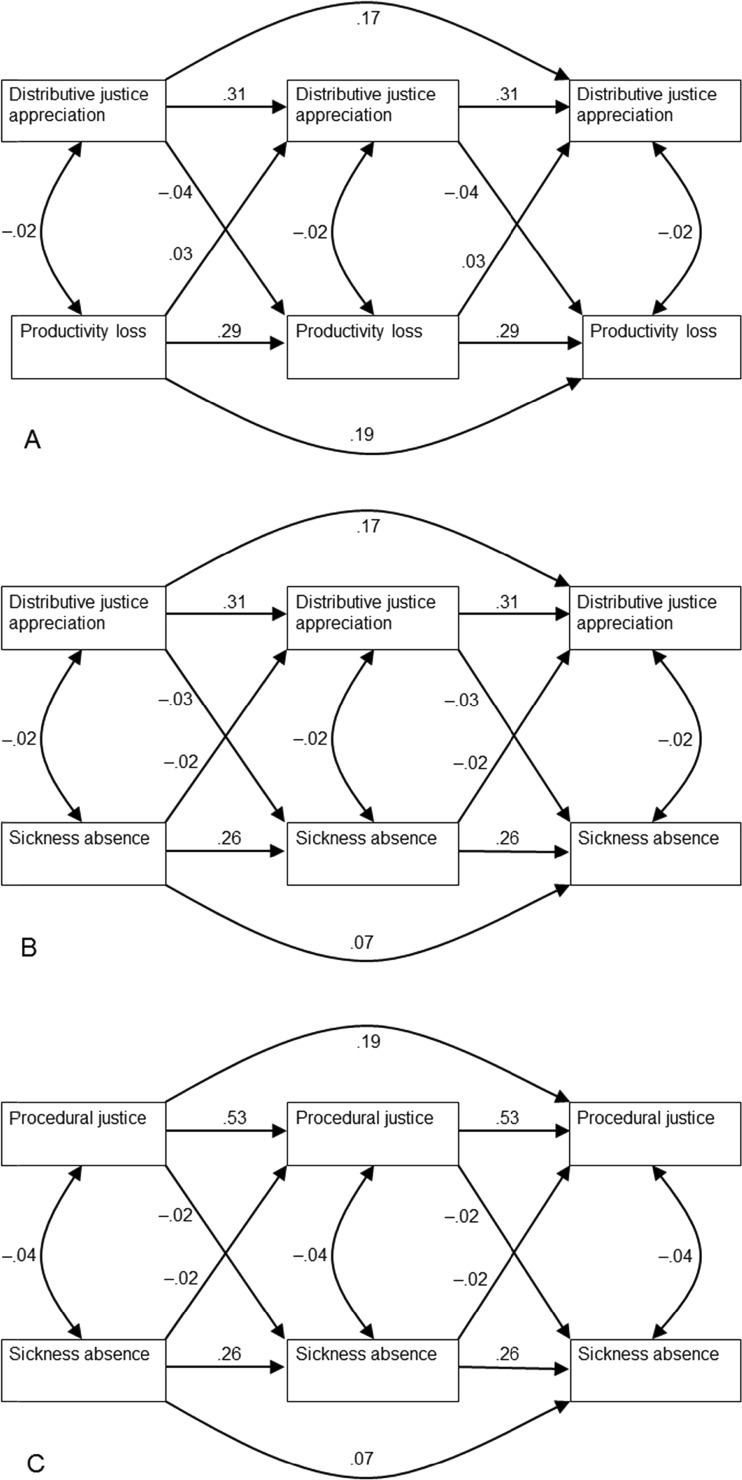
Three extracts from the reciprocal model (M5) with cross-sectional and longitudinal paths with standardized coefficients

The reciprocal relationships between productivity loss and distributive justice of appreciation are presented in more detail in Fig. 3a. The longitudinal patterns show that—in line with hypothesis 1a—distributive justice of appreciation contributed to lower productivity loss after correction for productivity loss in the previous year. On the other hand—in line with hypothesis 2a—productivity loss increased distributive justice of appreciation after correction for earlier distributive justice.

### Organizational Justice and Sickness Absence

Hypothesis 1b stated that organizational justice would lower sickness absence. As was the case for productivity loss, Fig. 2 shows that distributive justice of salary did not influence sickness absence (β = −0.01, *ns*). However, as predicted, both distributive justice of appreciation (β = −0.03, *p* < 0.01) and procedural justice (β = −0.02, *p* < 0.05) contributed to lower sickness absence a year later.

With regard to the reversed effects of sickness absence on organizational justice, no reversed longitudinal effects of sickness absence on distributive justice of salary were found (β = 0.00, *ns*). However, sickness absence lowered distributive justice of appreciation (β = −0.02, *p* < 0.05) a year later, which is in line with hypothesis 3b (*erosion*). For procedural justice, a similar reversed effect of sickness absence was found (β = −0.02, *p* < 0.01).

The reciprocal relationships between sickness absence and distributive justice of appreciation are presented in more detail in Fig. 3b. The longitudinal patterns show that—in line with hypothesis 1b—distributive justice of appreciation contributed to lower sickness absence after correction for sickness absence in the previous year. On the other hand—in line with hypothesis 3b—sickness absence reduced distributive justice of appreciation after correction for earlier distributive justice.

In Fig. 3c, the reciprocal relationships between procedural justice and sickness absence are shown. Procedural justice and distributive justice of appreciation showed identical patterns in the relationship to sickness absence: as hypothesized (hypothesis 1b), procedural justice lowered sickness absence in the following year, corrected for earlier sickness absence. Moreover, although not specifically hypothesized, sickness absence also lowered procedural justice corrected for earlier procedural justice.

### Demographic Variables

The standardized solution of the reciprocal model (M5) showed the following significant effects (gamma) of the demographic variables on organizational justice and on productivity loss and sickness absence. Women experienced higher distributive justice of both salary (γ = 0.04, *p* < 0.001) and appreciation (γ = 0.05, *p* < 0.001), but somewhat lower procedural justice (γ = −0.01, *p* < 0.05) than men. Moreover, women had more productivity loss (γ = 0.03, *p* < 0.05), and more sickness absence (γ = 0.04, *p* < 0.001) than men. Older employees experienced higher distributive justice of both salary (γ = 0.03, *p* < 0.001) and appreciation (γ = 0.02, *p* < 0.001) than younger employees. Higher educated employees experienced higher distributive justice of salary (γ = 0.03, *p* < 0.01) and higher procedural justice (γ = 0.03, *p* < 0.001) and were less absent from work (γ = −0.03, *p* < 0.001) than lower educated employees.

## Discussion

In the current longitudinal study with 2 years of follow-up, the relation between organizational justice and productivity loss and sickness absence was assessed. In line with the hypotheses, it was found that higher distributive justice of appreciation and higher procedural justice were related to lower productivity loss and lower sickness absence, when correcting for earlier productivity loss and sickness absence. This is a highly relevant result for organizations: a fair treatment of employees can prevent productivity loss and sickness absence, whereas lack of appreciation and unfair procedures may lead to productivity loss and sickness absence. These results augment earlier findings on the effects of organizational justice on depression, well-being, and sickness absence [13–16, 30–32] and more clearly show the relevance of organizational justice for older employees’ functioning at work.

Our second research question concerned the reversed effects of productivity loss and sickness absence on organizational justice. There was clear evidence that productivity loss and sickness absence are predictors as well as consequences of perceived organizational justice, although the normal effects of organizational justice on productivity loss and sickness absence were stronger than the reversed effects. It was found that productivity loss *increased* distributive justice of appreciation. This pattern of results is in line with our hypothesis 2a (*restore equity*) and the withdrawal perspective: inequity leads to productivity loss which restores equity due to the reduced investments in the job. If employees find that their investments (e.g., effort, results) are not appreciated adequately, they are likely to reduce these investments by becoming less productive in order to bring their investments in line with the appreciation received. The result is that equity is restored, but at a lower productivity and appreciation level, which is harmful for the organization.

For sickness absence, the reversed effects can be even more debilitating for an organization. In line with the erosion hypothesis 3b, high sickness absence reduced both procedural justice and distributive justice of appreciation, which could indicate that relationships at work may erode as a result of (long term) sickness absence. This could lead to a vicious circle in which lower organizational justice further increases sickness absence.

An interesting question is why both withdrawal behaviors have opposite effects on experienced distributive justice of appreciation. Perhaps, sickness absence has more negative social consequences than productivity loss. When experiencing health problems, coworkers may see presenteeism as an effort to still show constructive work behavior but may regard sickness absence as unnecessary withdrawal from work [9]. As a result, the reduction in appreciation may be stronger after sickness absence than after productivity loss when present at work, and stronger than the employee thinks is fair. This means that absence does not restore equity but rather erodes appreciation from others at work.

No direct longitudinal relationships between distributive justice of salary on the one hand and productivity loss and sickness absence on the other hand were found. This means that fairness of appreciation and procedural fairness are more important for maintaining a high productivity and reducing sickness absence than a fair salary. Moreover, additional analyses showed that in the present study an unfair salary did not increase productivity loss and sickness absence indirectly through feeling unfairly appreciated.

A number of strengths and limitations of the present study should be mentioned. The main strength is that the relationships between organizational justice, productivity loss, and sickness absence are examined in a longitudinal full panel design with three yearly measurements. This makes it possible to examine both the consequences and the antecedents of organizational justice. Although the effects of organizational justice on productivity loss and sickness absence were stronger than the reversed effects, our results indicate that productivity loss and sickness absence influence organizational justice as well. A second notable strength is the large and heterogeneous sample of older employees, which provides sufficient power for examining these longitudinal relationships.

A limitation of the study is that the longitudinal effects are small. This is in line with the more general finding that organizational fairness has only modest effects on withdrawal behaviors [4]. This means that the practical relevance of the found effects may be somewhat limited, although the vicious cycle found for sickness absence may reach substantial cumulative effects over the years. Moreover, as Zapf et al. [21] have argued, small effects are to be expected in longitudinal studies and we regard these small effects as meaningful because they are corrected for stability in the dependent variable, and for the longitudinal effects of other variables in the model, leaving relatively little systematic variance to be explained. Another reason for small effects is that the timing of measurements was not necessarily optimal for all respondents. Given that these measurements were a year apart, only generalized effects of organizational justice on sickness absence and productivity loss could be found.

A second limitation is that we used self-report measures of sickness absence and productivity loss. For sickness absence, there is evidence that self-reported absence is strongly correlated with registered absence, although employees may underestimate the number of absence days [33]. This means that self-reported sickness absence generally leads to valid conclusions in survey research. For productivity loss, future research should examine how individuals’ own perceptions of productivity loss correspond to the perception of their supervisors or colleagues.

Another limitation lies in our measures for organizational justice. Although we believe that our measures are highly valid, they differ from more often used measures, such as the Colquitt scale [34]. Our study focused on distributive and procedural justice and did not include a measure of interactional or informational justice [35]. However, our measures of procedural justice and distributive justice of appreciation both convey information about the quality of the relationship with the supervisor, i.e., interactional justice [7, 8]. Our measures of distributive justice of salary and appreciation were strongly related over time and enhanced each other, as was the case for distributive justice of appreciation and procedural justice. This strengthens the idea that there may be a single common (second order) factor of organizational justice that underlies most of its effects [36], which is the feeling of being fairly treated [8]. Adding a measure of interactional justice would probably be superfluous and would further increase the overlap between the justice measures.

We chose our measures of distributive justice mainly because we wanted to stay close to the original conceptualization of distributive justice in comparison to coworkers [5], including both under-benefit and over-benefit, rather than the more general fairness of outcomes that is often measured [35]. Nevertheless, we did not differentiate between over-benefit (receiving too much appreciation or salary) and under-benefit (receiving too little) in the present study. This is in line with equity theory, which predicts that both over-benefit and under-benefit may have negative consequences [5]. However, at work, perceiving under-benefit is more likely and probably has more negative consequences than perceiving over-benefit [37]. Additional analyses showed that in our study under-benefit was indeed more prevalent than over-benefit and that under-benefit had stronger relationships with productivity loss and sickness absence.

The present study specifically focuses on productivity loss and sickness absence of employees aged 45 years and older. This is a very relevant group of employees given the recent necessity to remain working until a higher age. However, it remains an empirical question whether the relationships shown in this study will be similar or different among younger employees. We believe that fair treatment will be important for employees at all ages, although the specific responses to different kinds of injustice may differ for younger and older employees. For example, younger employees may be more likely to change jobs in the face of injustice, whereas older employees may be more likely to retire early [38]. Future research should include both older and younger employees and compare their responses to organizational justice.

We conclude that there is strong evidence from past research [4, 13] that organizations can increase the health and well-being of their personnel by investing in organizational justice. The present study suggests that organizations may also reduce productivity loss and sickness absence among their older employees by such investments. A fair treatment that takes the interests of employees into account and a fair appreciation that is in line with the investments and results of employees are important for a motivated and productive workforce. Organizations are therefore likely to benefit economically from investments in a just organizational climate.
